# Advancing Science through Conversations: Bridging the Gap between Blogs and the Academy

**DOI:** 10.1371/journal.pbio.0060240

**Published:** 2008-09-23

**Authors:** Shelley A Batts, Nicholas J Anthis, Tara C Smith

## Abstract

Blogs have stormed the Internet, providing an interactive medium for rapid and wide-reaching information dispersal. But is there a place for blogs in academia?

Scientific discovery occurs in the lab one experiment at a time, but science itself moves forward based on a series of ongoing conversations, from a Nobel Prize winner's acceptance speech to collegial chats at a pub. When these conversations flow into the mainstream, they nurture the development of an informed public who understand the value of funding basic research and making evidence-based voting decisions. It is in the interests of scientists and academic institutions alike to bring these conversations into the public sphere.

With 39% of American Internet users reading at least one of the over 112 million blogs on the Internet, blogs represent a means of information dispersal with unprecedented power [1]. Science blogs have carved out a small but influential niche, with an estimated number of over 1,200 [2]. One of the most popular science blogs, Pharyngula, began as a classroom teaching tool and now logs over 1.5 million visitors and thousands of new comments each month (http://scienceblogs.com/pharyngula/). The blog aims to provide a universal, interactive rallying point for understanding and discussing evolutionary development, and is led by a professor of biology at the University of Minnesota, Morris. Pharyngula's popularity demonstrates how a small blog can rapidly skyrocket in readership and should encourage those concerned about science literacy in America.

Furthermore, blogs can have a substantial impact on traditional academia by providing a quick forum for public peer review of research. For example, in 2005 Reed Cartwright, postdoctoral fellow and blogger at *De Rerum Natura* (http://dererumnatura.us/), disagreed with the conclusions of a paper in *Nature* [3] and posited what he argued was a more probable interpretation of the data on his blog [4]. University of Washington researcher Luca Comai was about to publish a letter to the editor arguing for the same alternate hypothesis when he read Cartwright's blog, and realized he had been beaten to the punch. In the end, the blogger and researcher made their case in a jointly authored paper in *Plant Cell* [5].

Because many science bloggers are practicing scientists or experts in their field, they can provide a unique educational bridge between academia and the public and distill important experimental findings into an accessible, interactive format. Yet academic institutions have been slow to appreciate blogs as valuable mediums for facilitating scholarly discussion, illustrated by the lack of institutional blogs or blogs by established academics. It is true that few quality-control or vetting mechanisms exist to help readers evaluate a blog, which typically earns its reputation based on the blogger's credentials and reader feedback. Yet both academic institutions and blogs aim to engage and educate the public and advance scientific knowledge and discussion. By combining the credibility of institutions—trusted gate-keepers for scientific truth—with the immediacy and networking infrastructure of blogs, we believe that these shared goals can be better served with benefits to both partners.

We propose a roadmap for turning blogs into institutional educational tools and present examples of successful collaborations that can serve as a model for such efforts. We offer suggestions for improving upon the traditionally used blog platform to make it more palatable to institutional hosts and more trustworthy to readers; creating mechanisms for institutions to provide appropriate (but not stifling) oversight to blogs and to facilitate high-quality interactions between blogs, institutions, and readers; and incorporating blogs into meta-conversations within and between institutions.

## Building the Foundation from the Bottom Up

**Figure 1:**
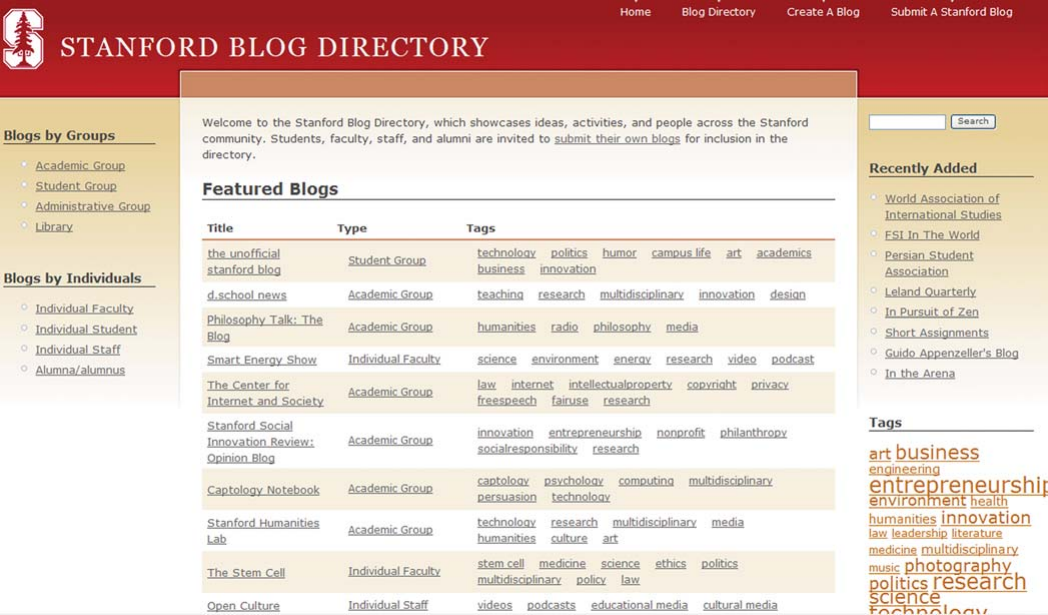
Screenshot of the Hub of the Stanford Blog Directory A central, university-sponsored hub provides links to all institution-affiliated blogs that petition to join.

The easiest way for an academic institution to test the blogging waters is to showcase existing blogs written by faculty, students, or alumni. To begin, an institution can either modify its Web presence to include a hub for intra-institutional blogs, or aggregate links to blogs on similar topics. For example, the Stanford Blog Directory, which includes links to internal and external blogs by Stanford-associated faculty, staff, students, and alumni (Figure 1), lists over 150 existing blogs indexed by blogger-chosen keywords and blogger affiliation (http://blog.stanford.edu/). The directory was spearheaded by Stanford Director of Internet Media Outreach Ian Hsu, who hoped that creating a common home for Stanford blogs might encourage faculty to use blogs as a medium for communicating interesting research to lay readers as well as their peers. (For inclusion in the directory, Hsu requires only that a blog be written by a member of the university and not violate Stanford's information technology terms.) This setup benefits both the institution, which gets free publicity for its researchers' work, and academic bloggers, who have a built-in readership funneled straight from the institution's Web page. At this point, however, the Stanford Blog Directory has no mechanisms through which readers can engage in an academic conversation on the hub itself, except for a “featured blogs” category. To create a more dynamic conversation, the site could add a centralized search feature, updated links to the latest posts, or hubs linking blog posts about recent research to news stories and the journal article itself.

Universities can also take a bottom-up approach by encouraging blogs centered around an institute, as in the case of Rudd Sound Bites, the group blog of Yale University's Rudd Center for Food Policy and Obesity Research (http://www.ruddsoundbites.typepad.com/). The blog, though not officially sponsored by the university, is written by Center faculty and staff, with submitted entries going to a central manager for minor editing and approval. The ChemTools blog listing, which provides links to the blogs of chemists and biochemists at the University of Southampton, is likewise self-managed and independent of the university (http://chemtools.chem.soton.ac.uk/projects/blog/). The Berkeley Lab Energy and Environmental Research Blog, a joint blog between the US Department of Energy and Berkeley Lab at the University of California, provides a model for a partnership between a government agency and an institution of higher learning (http://bleer.lbl.gov/). The blog provides a mechanism to bridge a shared mission; in this case, to report news and research about climate shift and energy, and their impact on policy. The Oxford Internet Institute, which studies the impact of online technology, hosts a network of blogs written by students and fellows at the institute and allows considerable intellectual freedom (http://www.oii.ox.ac.uk/). Although all blog posts appear on the main page, which is on a University of Oxford URL, anyone affiliated with the institute can feed their blog onto the institute blog without moderation. Appropriately, blogs are prominent features on the Web site for the Lab for Social Computing at the Rochester Institute of Technology and provide an accessible interface for readers to learn about the mission of the center (http://social.it.rit.edu/).

Some bloggers see institutionally based blogging as a way to advance an institution's academic mission. For Roger Pielke, Jr., blogger at Prometheus (http://sciencepolicy.colorado.edu/prometheus/), a science policy blog affiliated with the University of Colorado Center for Science and Technology Policy Research, “blogging has become really inseparable from academic life in a lot of respects.” Pielke says he acquired numerous collaborators via the blog, and authored several publications, including two recent high-impact papers published this year that were directly informed by discussions on Prometheus [6,7]. Prometheus gets about 25,000 visits each month by bringing in outside voices and providing an outlet for laypeople to ask institute members questions. Pielke believes that grounding the blog in an academic institution improves its legitimacy and reach.

## Bridging the Gap from the Top Down

Institutions might also try a “top-down” approach, following the model of the Massachusetts Institute of Technology's (MIT) Technology Review (http://www.technologyreview.com/Blog/), which has online, print, editorial, and blog content for laypeople, scientists, and alumni. When deciding to add expert blogs to their Web presence, Technology Review actively recruited current MIT assistant professor Ed Boyden to write a recurring blog on topics he thought were both interesting and could benefit from the fast and brutally honest discussion that the Web can provide. Although Technology Review posts are read by editors before being published, Boyden attests that his posts have never been substantially changed. He states that “blogging allows me to fulfill the academic mission in another way which narrowly focused journals cannot.”

Scholarly journal articles are not intellectually accessible to most of the population, and are often behind an expensive pay-wall. Conversely, science blogs are freely accessible, interactive, and are generally written for a lay audience. Although only a small percentage of the 38% of 12- to 17-year-olds who read blogs may be reading science blogs, blogs clearly have the potential to reach an age group where excitement about a future career in science could be ignited [8]. An excellent example of an educational, fun, and accessible science blog is The Panda's Thumb, where evolutionary biologists tackle questions about evolution in easy-to-understand ways, and science teachers are an important part of their audience (http://www.pandasthumb.org/).

In considering a top-down initiative, the institution's representative and the potential blogger should have a frank discussion about the expectations of the blog's format, content, goals, commenting policies, time commitment, level of acceptable bias, what perks or drawbacks the author might experience, and how the institution plans to provide oversight to the blog. While Technology Review does ask that a faculty blogger sign a contract, it is flexible and serves mainly to outline common goals and potential (modest) financial rewards to the blogger.

## Providing Quality Control

How an institution gauges the success of the blog will depend on many factors. In the blogosphere, peer evaluation is often carried out informally, as readerships—and reputations—are built largely upon links from other blogs. As a blog's clout grows, so does its Technorati rank (http://www.technorati.com/) and precedence in related Google searches. These in turn lead back to the institution, which would not have access to some of these powerful avenues for traffic without blogs. Institutions may wish to implement more formal vetting mechanisms, however, such as periodic review by institutional moderators or peer review by official committees of blog-literate individuals, established scientists, and bloggers. Institutions might use one of a variety of mechanisms to confer a visible token of this review—such as a “blog badge”—in order to both reward quality bloggers and help readers identify trusted blogs. A blog badge is simply a small picture or icon that is prominently featured on the blog and represents an award or achievement. Such badges are usually given as awards (such as the “Weblog Awards” [http://weblogawards.org/] or the “MedBlog Awards” [http://www.medgadget.com/archives/2007/12/the_2007_medical_weblog_awards_sponsored_by_scrubsgallerycom.html]), and are awarded to particular outstanding blogs in a variety of categories, such as “Best Group Blog,” “Most Informative,” and “Best Translation of Published Research.” Traditional blogging awards are conferred by a committee who invites submissions until a deadline, reviews them, and then posts the winners on their Web site. The winners can then download the badge to post on their blog. Institutions might find it useful, and bloggers might find it motivating, if institutional blog badges were conferred for particularly insightful posts or as a token for passing their test or review periods. Accumulating these badges would be a public and official way for the institution to reward and validate the blogger, while conferring authority to the blog by letting readers know it has met the criterion for institutional peer review. While it is true that this system might be abused by sites posting the badge without actually winning the award or passing review, badge recipients are always listed on the awards Web site and it is difficult to fool the countless eyes of the ever-observant Internet for long.

**Figure 2:**
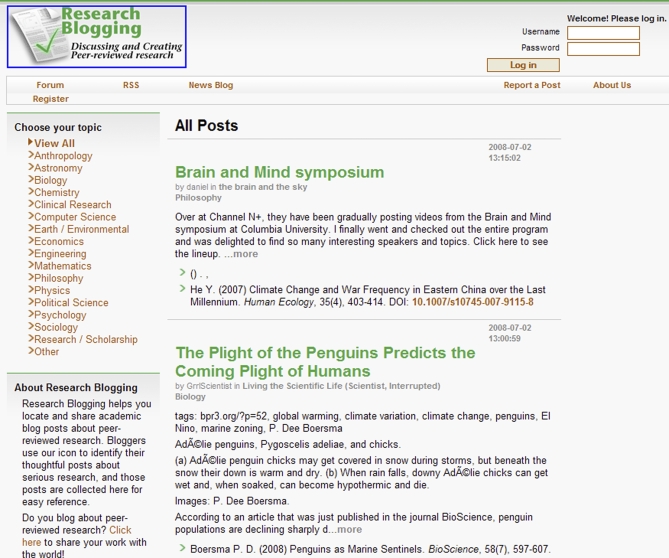
Screenshot of the Home Page of ResearchBlogging.org Blog posts that embed the project's coded badge are aggregated at this central site, with the peer-reviewed paper clearly cited.

Bloggers can also use badges to self-identify academically relevant blogging. ResearchBlogging.org, for example, automatically aggregates only blog posts about peer-reviewed research (Figure 2; http://researchblogging.org/) by searching preregistered blogs for a piece of code that's included in an automatically generated reference bloggers can place in posts about peer-reviewed research. The site also offers an optional icon for bloggers to identify these posts to their readers. Since starting up in January 2008, ResearchBlogging.org has approved over 400 science blogs. To be included on the site, a blog must demonstrate to the site's organizers via a submitted form that it regularly produces posts that would meet the criteria for use of the icon. Once included, it's then up to the blogger to decide which posts meet a set of detailed guidelines for use of the icon. Dave Munger, the initiative's cofounder and president, describes the project as largely self-regulating. Readers are encouraged to report abuses of the icon, which may lead to the permanent removal of a blog. This happened in the case of an anti-evolution blog that had coopted the system, attempting to use the icon while posting non-peer-reviewed “studies” about creationism. A reader reported the abuse, and after a review by the moderators, the blog was denied future use of ResearchBlogging.org. This system illustrates that with a bit of technical savvy, a few guidelines, and an involved readership, the self-regulating style of the blogosphere can be harnessed in new ways that could prove useful for institutional science outreach.

Nearly all existing blogging initiatives have started from the bottom up, rather than under the guidance and authority of the institution. This may be reflective of the free-flowing, decentralized nature of blogs themselves. But if groups of bloggers were to create their own initiatives and then seek institutional recognition, they might be able to engage in conversations about science on their own terms while continually proving to the institution—as they already strive to prove to their readers and peers—that the conversations they are engaging in are worthwhile. As part of a thriving online scientific community sustained by unprecedented connectivity, immediacy, interactivity, and reach, bloggers can help academic institutions take advantage of a powerful tool for the dissemination of scientific information and facilitation of conversations about science. In addition to providing a bridge between science communication and the public, institutional blogs could facilitate collaborations of scientists separated by distances as small as a few buildings or as large as the Pacific Ocean. We believe that the ideal relationship, be it blogger- or institution-initiated, is one where blogs are used as forges for developing ideas and forums for the discussion of accurate, interesting, and up-to-date scientific information, with the institution creating links between blogs while conferring authority. By initiating frank and open-minded conversations about shared goals, blogs and institutions can work together to advance the quality and scope of the ongoing global conversation about science we all participate in and depend upon.
